# The association between asthma and atrial fibrillation: systematic review and meta-analysis

**DOI:** 10.1038/s41598-023-50466-w

**Published:** 2024-01-26

**Authors:** Beatriz Nogueira-Garcia, Mariana Alves, Fausto J. Pinto, Daniel Caldeira

**Affiliations:** 1 Serviço de Cardiologia, Hospital Universitário de Santa Maria – CHULN, Lisbon, Portugal; 2 Serviço de Medicina III, Hospital Pulido Valente, CHLN, Lisbon, Portugal; 3 Laboratório de Farmacologia Clínica e Terapêutica, Faculdade de Medicina, Universidade de Lisboa, Lisbon, Portugal; 4 Instituto de Medicina Molecular, Faculdade de Medicina, Universidade de Lisboa, Lisbon, Portugal; 5 Centro Cardiovascular da Universidade de Lisboa – CCUL (CCUL@RISE), CAML, Faculdade de Medicina, Universidade de Lisboa, Av. Prof. Egas Moniz, 1649-028 Lisbon, Portugal; 6 Centro de Estudos de Medicina Baseada na Evidência (CEMBE), Faculdade de Medicina, Universidade de Lisboa, Lisbon, Portugal

**Keywords:** Cardiology, Medical research, Risk factors

## Abstract

Respiratory disease and atrial fibrillation (AF) frequent coexist, but the risk of AF among asthma patients is less characterized. Growing evidence suggest that AF shares with asthma a systemic inflammation background and asthma treatments, such as beta agonists, have been associated with increased risk of cardiac arrhythmias. The aim of this systematic review was to assess the risk of AF in patients with asthma in observational studies. We search for longitudinal studies reporting AF outcome in asthma and control patients through MEDLINE, Cochrane Central Register of Controlled Trials and EMBASE. Pooled estimates of odds ratios (ORs) and 95% confidence intervals (CIs) were derived by random effects meta-analysis. Heterogeneity was assessed using the I2 test. The risk of bias of individual studies was evaluated using the ROBINS-E tool. The study protocol was registered at PROSPERO: CRD42020215707. Seven cohort/nested case–control studies with 1 405 508 individuals were included. The mean follow-up time was 9 years, ranging from 1 to 15 years. Asthma was associated with a higher risk of AF (OR 1.15. 95% CI 1.01–1.29). High heterogeneity (I^2^ = 81%) and overall “serious” risk of bias, lead to a very low confidence in in this result. Asthma was associated with an increased risk of AF. However, the high risk of bias and high heterogeneity reduces the robustness of these results, calling for further high-quality data.

## Introduction

Atrial fibrillation (AF) is a supraventricular arrhythmia characterized by uncoordinated atrial electrical activation. It is the most common sustained arrhythmia worldwide and is associated with significant patients’ mortality and impacts quality of life^1,2^. However, AF can be silent, and the first clinical manifestation may be a stroke if this arrhythmia is not diagnosed before^3,4^. Therefore, it is important to recognize which are the clusters of patients that are more prone to develop AF to plan screening strategies.

Respiratory diseases and AF frequently coexist but the relationship with asthma is not well characterized^5,6^. Proposed mechanisms include an inflammatory pathway, particularly in obstructive sleep apnea which is one of the respiratory diseases most commonly associated with AF. Other respiratory diseases such as asthma are starting to show an association with AF^7^. It is now recognized that pathophysiology of AF is extremely heterogeneous^8,9^, sharing with asthma an inflammatory pathway. Systematic inflammation may conduct to atrial electrophysiology and structural remodelling, leading to increase vulnerability to AF^10^. Leukotrienes—inflammatory mediators produced in leukocytes—have systematic effects and their receptors are highly expressed in the heart^11,12^. Furthermore, asthma treatments, such as beta-2 adrenergic agonists, and airway obstruction with hypoxemia have been associated with increased risk of cardiac arrhythmias^13,14^, but some doubts still exist regarding this link.

The aim of the present systematic review was to evaluate the available evidence regarding the risk of atrial fibrillation in patients with asthma.

## Methods

We performed a systematic review and meta-analysis following the PRISMA (Preferred Reporting Items for Systematic Reviews and Meta-Analysis)^15^ and “The Meta-analysis of Observational Studies in Epidemiology”^16^ recommendations. The study protocol was registered with PROSPERO (CRD42020215707).

### Data sources and search strategy

Potentially eligible studies were identified through an electronic search of bibliographic databases from inception to December 2020 (MEDLINE through PubMed, Cochrane Central Register of Controlled Trials and EMBASE). The search methods used are summarized in Supplementary Table 1. Additionally, we searched for relevant data by checking the reference lists of included studies. No dates or language restrictions were applied.

### Eligibility criteria

For the purposes of our systematic review all longitudinal (prospective or retrospective) studies reporting atrial fibrillation outcome in both patients with asthma—defined by clinical signs/symptoms and/or functional tests, administrative codes or as defined by the physician/investigator—and matched controls were considered eligible.

### Study selection and data extraction

Two authors (BG and MA) independently screened the title and abstracts of the citations retrieved in the electronic database search^17^. The full-text reports of all potentially relevant studies were obtained and the authors independently selected studies to be included in the review according to the predefined inclusion criteria. Doubts and disagreements were resolved by consensus. Reasons for the exclusion of articles were recorded.

Whenever available, data extracted included: study design, location, period of study, patient and control population characteristics, outcomes of interest and the adjustments of estimates.

### Outcome

Atrial fibrillation was the primary outcome. It was defined as a supraventricular tachyarrhythmia with uncoordinated atrial electrical activation and consequently ineffective atrial contraction, with abnormal ECG activity (absence of P waves and irregular R–R intervals)^18^. Diagnosis made by the patient’s physician or corresponding administrative code were also acceptable for the definition of the patient’s condition.

### Study-level risk of bias and meta-biases

Each study was evaluated independently by two authors (BG and MA) in each of the domains of bias contained in the ROBINS-E tool, accordingly to the algorithm^19^. Then, the overall risk of bias judgement was performed. Publication bias was evaluated through funnel plot evaluation and Egger test.

### Assessment of confidence in the cumulative evidence

The evaluation of primary outcomes was performed using the Grading of Recommendations Assessment, Development and Evaluation (GRADE) framework regarding the study design, study quality, consistency, and directness^20^. The pooled evidence was then classified as having very low, low, moderate, or high confidence.

### Data synthesis

We used STATA 17.0 to derive forest plots and to perform pooled analysis and related tests. Random-effects meta-analysis was performed with the DerSimonian-Laird model to estimate pooled odds ratio (OR) and 95% confidence intervals (95% CIs). Heterogeneity was defined as *p* value < 0,10 in the Chi-square test and the magnitude was reported through the I^2^ metric and was considered substantial if I^2^ > 50%^21^. Meta-regression was performed to evaluate the impact of age and follow-up.

## Results

### Included studies

**Figure 1:**
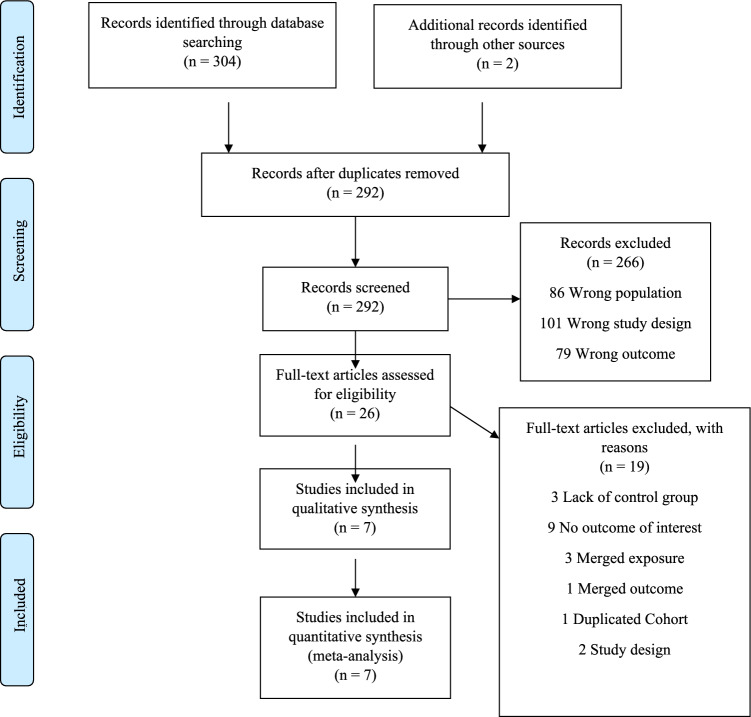
Flow diagram showing the study selection process.

The search of the electronic databases yielded 292 studies after removal of duplicates. From those, 266 were excludes after titles and abstracts screening and full-text assessment was performed for 26 articles. Following our inclusion and exclusion criteria, we were able to include 7 studies for analysis (Fig. 1). The main reasons for excluding studies were lack of control group and no assessment of the outcome of interest.

### Description of the studies

All studies had a prospective/retrospective cohort design^7,22–27^. Four studies were conducted in Europe, two in America and one in Asia. The mean follow-up time was 9 years, ranging from 1 to 15 years. Overall, 1,405,508 patients were included. Table 1 shows the main study characteristics.

**Table 1:** Main characteristics of the included studies.

Study	Study design	Mean follow-up (years)	Location	Data source	Asthma patients			Control population			Matching	AF dignosis method
					Number	Mean (SD) age, y	Male gender (%)	Number	Mean (SD) age, y	Male gender (%)		
Carter, 2019	Prospective cohort/Nested case–control	12.3	United Kingdom	7 National Health Service Hospitals	60,424	48.5 (20)	36.7%	302,120	48.6 (20)	36.7%	Age and gender	ICD-10 and OPCS-4
Cepelis, 2018	Prospective cohort/Nested case–control	15.4	Norway	Survey-based Nord-Trondelag Health study	3934	NR	NR	48,606	NR	NR	NR	ICD-10 and medical record review
Chamberlain, 2017	Prospective cohort/Nested case–control	6.3	Minnesota USA	Rochester Epidemiology Project, a records-linkage system	715	73.6 (13.8)	48.6%	715	72.7 (13.5)	48.6%	Age, gender and calendar year of diagnosis	ICD-9 codes or ECG
Chan, 2014	Retrospective cohort/Nested case–control	7	Taiwan	Taiwan National Health Insurance database	3975	71.5 ( 13.1)	44%	13,539	71.1 (13.7)	50%	Age, gender, comorbidity, and cohort entry date	ICD-9- and ECG/Holter monitoring
Jani, 2018	Prospective cohort/Nested case–control	7	United Kingdom	Community cohort participants (UK Biobank)	4238	NR	NR	496,654	NR	NR	NR	Self reported
Martín-Pérez 2016	Prospective cohort/Nested case–control	2.67	United Kingdom	The Health Improvement Network	1041	75.78 (8.9)	55%	3448	75.78 (8.9)	55%	Age (within 1 year) and gender	Read classification
Tattersall 2020	Prospective population cohort/Nested case–control	12.9	USA	Multi-Ethnic Study of Atherosclerosis	647	60.4 (10)	66.4%	5968	62.2 (10.2)	48.5%	NR	ECG, ICD9/ICD9-CM code, Medicare data

Abbreviations: AF, atrial fibrillation; ECG, electrocardiogram; ICD, International Classification of Disease; NR, not reported; OPCS – 4 Office of Population Censuses and Surveys Classification of Interventions and Procedures.

Many studies did not report previous cardiovascular risk factors of the participants included (Supplementary Table 2). Asthma diagnosis methods were essentially based on codification systems and patient report. Almost all did not mention asthma severity and the type of medications for asthma control (Supplementary Table 3). Outcome adjustment for cardiovascular risk factors was present in only few studies (Supplementary Table 4).

### Risk of bias

The risk of bias is serious mainly due to significant bias in the measurement outcome domain (Table 2). Diagnosis method of asthma vary from self-reported to detection through codification systems. In terms of AF diagnostic, it was also verified an important heterogeneity, being often based on codification systems but in some studies an ECG or Holter monitoring was required. Bias in selection of participants was classified as serious in two studies—Carter et al.^22^ and Marín-Pérez et al.^28^—since the participants are part of a hospitalized and heart failure population, respectively, which can influence the risk and enhance AF diagnosis. On the other hand, in these two studies the risk in measurement outcome was accepted as being low once this kind of populations usually performed ECG regularly.

**Table 2:**
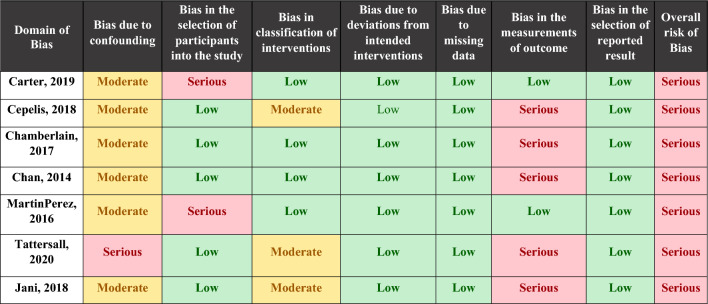
Risk of bias.

The risk for each study included in the meta-analysis was assesses following the algorithm ROBINS—E.

### Outcome: risk of atrial fibrillation

**Figure 2:**
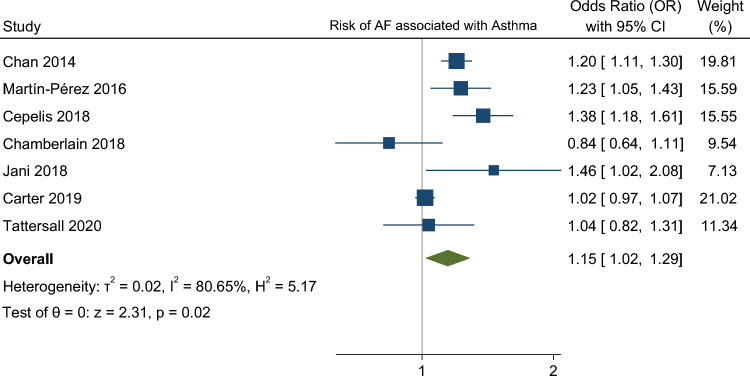
Forest plot for risk of atrial fibrillation associated with asthma.

**Figure 3:**
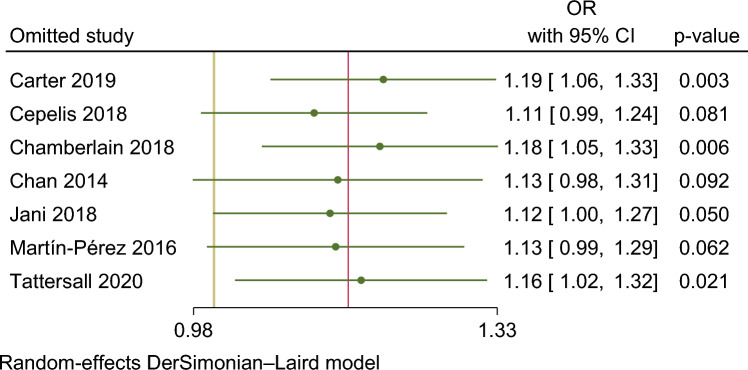
Sensitivity analysis evaluating the result of the meta-analysis with exclusion of each single study (leave-one-out analysis).

**Figure 4:**
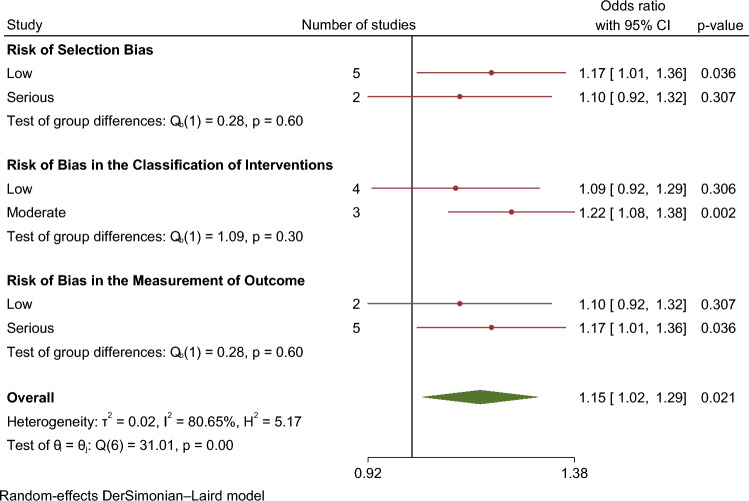
Results of subgroup analysis according to the evaluation of risk of bias.

In our meta-analysis asthma was associated with a higher risk of AF (OR 1.15. 95% CI 1.01–1.29) (Fig. 2). The analysis showed very high statistical heterogeneity (I^2^ = 81%). A sensitivity analysis evaluating the result of the meta-analysis with exclusion of each single study was performed and the outcome remain consistent regarding the direction and magnitude of the effect, however the significance was lost with the exclusion of the studies Cepelis et al., Chan et al. and Martin-Pérez et al. which can be considered a robustness indicator (Fig. 3). Subgroup analysis according to the evaluation of risk of bias was also performed and the results between low and moderate or serious risk of bias regarding different domains, such as selection bias, classification of interventions and measurement outcome, were quite consistent (Fig. 4).

Meta regression results analyzing the effect of age and follow in the results did not find any significant estimates (*p* = 0.326 and *p* = 0.587, respectively) (Supplementary Fig. 1). Only few studies reported data according to asthma severity and asthma treatment.

The evaluation of the funnel plot and Egger regression test (*p* = 0.89) result did suggest the existence of publication bias (Supplementary Fig. 2).

### Assessment of confidence in cumulative evidence

Applying GRADE criteria, the confidence in the evidence is very low, being the downgrading reasons mainly due to risk of bias and imprecision—Supplementary Table 5.

## Discussion

Our analysis showed that asthma was significantly associated with a higher risk of AF, however with very low certainty according to GRADE criteria.

It is now recognized that pathophysiology of AF is complex and probably in association with systemic disease^9,29^. The initiation and maintenance of this supraventricular arrhythmia can be the result of the interaction between a trigger and the substrate, induced by an electrical and structural remodeling^8^. Emerging evidence suggest mechanisms that goes beyond the atrium, sharing with asthma an inflammatory pathway. Inflammation represents a trigger of AF and is also implicated in its perpetuation^10^. Different inflammatory cytokines mediate function of ion channels and atrial remodelling^30,31^. Important mediators of inflammation such as C reactive protein and interleukin-6, have been found to be high in patients with AF, and even their influence on the success of the AF ablation have been shown^32,33^.

Furthermore, asthma treatments, such as beta 2-agonists and corticosteroid therapy, in different ways can modulate the risk of cardiac arrythmias^34^. The beta 2-adrenergic receptor agonists, such as salbutamol or formoterol, have a positive chronotropic effect and also decrease the atrioventricular nodal, atrial, and ventricular refractoriness which can enhance the risk of both supraventricular and ventricular arrhythmias^35^. In the other hand, corticosteroids were found to be beneficial for the prevention of atrial fibrillation occurrence in patients undergoing cardiac surgery and AF ablation procedures, probably due to its anti-inflammatory activity^36,37^.

There is suggestion that the severity of asthma as a surrogate of inflammation and beta2 adrenergic agonists use can influence the incidence of AF. However, most of the studies included in our analysis did not report patients’ asthma severity or medication used. In the few studies reporting such data there was a higher risk for AF in participants with uncontrolled asthma (HR 1.74 [95%CI 1.26–2.42]) (Cepelis et al.^25^ study); persistent asthma, defined as asthmatics requiring controller medications, but not intermittent asthma was associated with a 1.5-fold increase in risk of AF in Tattersall et al.^7^; in Chan et al.^24^ bronchodilator therapy—corticosteroid and non-corticosteroid—was associated with an increased risk for AF (OR 2.13; 95% CI 1.226–3.701, *p* = 0.007 and OR 2.849; 95% CI 2.48–3.273, *P* < 0.001, respectively). The association between AF and the use of inhaled corticosteroids is still unclear and a possible explanation for these results is the relationship between these drugs use and asthma severity.

The strength of association between asthma and AF might be weaker due to the statistical and clinical heterogeneity among the included studies as well as the risk of bias which preclude robust conclusion. More evidence is needed to establish robustly this relationship, which can be of tremendous value concerning identification of individuals at higher risk in the community and, therefore, improving prevention and screening programmes for early AF detection and stroke prevention. These studies should be able to provide further evidence about the magnitude of the relationship between asthma and drugs used in the AF outcome.

## Limitations

Our systematic review with meta-analysis has some limitations. The diagnosis of asthma was heterogenous in the included studies, varying from self-reported and codification systems and atrial fibrillation assessment was also variable (ECG or Holter monitoring and mainly based on codification system and patients’ reports) which represents a potential source of bias. The period of follow-up ranged from 1 to 15 years (mean follow-up time 7 years), which can influence the main outcome. One of the major drawbacks of most of the studies as well of this systematic review is that most of risk factors for AF were not adequately characterized which underpowers further exploratory analyses that were not performed due to lack of data (Supplementary Table 2).

The low confidence in the generated evidence was recognized by the GRADE assessment where the risk of bias due to confounding and risk the of bias due to the assessment and measurement of outcomes have an important role, and should be acknowledged in the planning of future studies.

Nevertheless, most of the outcomes were adjusted at least to some of these potential confounders, such as smoking, alcohol consumption, diabetes, lipid profile, arterial hypertension, obesity, and other cardiovascular diseases (Supplementary Table 4).

## Conclusion

The risk of AF associated with asthma patients was significantly increased. However, more evidence is necessary to support these association and clarify etiologic mechanisms. Identifying individuals at higher risk of developing AF could facilitate screening programmes and earlier diagnose. Based on our data it is wise to recommend special attention in clinical evaluation of asthma patients, including a systematic revision of symptoms like palpitations, dyspnea and fatigue, as well as opportunistic evaluation of the pulse regularity, to prompt ECG recording (e.g. 12-lead ECG or Holter monitoring).

### Supplementary Information

Supplementary Information 1.

Supplementary Information 2.

## Data Availability

The datasets generated and/or analysed during the current study are public. However, any data can be provided from the authors upon reasonable request by contacting Prof. Daniel Caldeira (dgcaldeira@hotmail.com), the corresponding author.
