# Supplementary material for: The association between asthma and atrial fibrillation: systematic review and meta-analysis
Source: Sci Rep. 2024 Jan 26;14:2241. doi: 10.1038/s41598-023-50466-w (PMC10817980; doi:10.1038/s41598-023-50466-w)
Supplement: Supplementary file 2 — Supplementary Information 2. [file 41598_2023_50466_MOESM2_ESM.docx]

**Supplementary data**

The association between asthma and atrial fibrillation: systematic review and meta-analysis.

Corresponding author

Professor Daniel Caldeira

Centro Cardiovascular da Universidade de Lisboa - CCUL, Faculdade de Medicina, Universidade de Lisboa, Portugal. Av. Prof. Egas Moniz, Lisboa 1649-028, Portugal; dgcaldeira@hotmail.com

**Index**

Supplementary Figure 1: results of the metaregression, 9

Supplementary Figure 2: : Funnel plot for the AF risk in patients with asthma, 10

Supplementary Table 1 – Search Methods, 3

Supplementary Table 2. Main population characteristics, 4

Supplementary Table 3. Severity and therapy of asthma patients, 6

Supplementary Table 4. Atrial Fibrillation Outcome, 8

Supplementary Table 5. Summary of findings according to GRADE criteria (Grading of Recommendation, Assessment, Development and Evaluation)., 11

**Supplementary Table 1 – Search Methods**

| # | Searches |
| --- | --- |
| 1 | exp asthma/ |
| 2 | (antiasthma$ or anti-asthma$).mp. |
| 3 | exp Respiratory Sounds/ |
| 4 | wheez$.mp. |
| 5 | Bronchial Spasm/ |
| 6 | bronchospas$.mp. |
| 7 | bronchoconstrict$.mp. |
| 8 | exp Bronchoconstriction/ |
| 9 | Bronchial Hyperreactivity/ |
| 10 | Respiratory Hypersensitivity/ |
| 11 | 1 or 2 or 3 or 4 or 5 or 6 or 7 or 8 or 9 or 10 |
| 12 | atrial fibrillation.af. |
| 13 | atrial flutter.af. |
| 14 | (Atrial adj1 Fibrillat*).af. |
| 15 | (Auricular adj1 Fibrillat*).af. |
| 16 | auricular flutter.af. |
| 17 | exp atrial fibrillation/ |
| 18 | exp atrial flutter/ |
| 19 | 12 or 13 or 14 or 15 or 16 or 17 or 18 |
| 20 | 11 and 19 |
| 21 | exp animals/ not humans.sh. |
| 22 | 20 not 21 |

**Supplementary Table 2. Main population characteristics**

|  | Carter  2019 | | Cepelis  2018 | | Chamberlain  2018 | | Chan 2014 | | Jani 2018 | | Martin-Perez 2016 | | Tattersall 2020 ^a)^ | |
| --- | --- | --- | --- | --- | --- | --- | --- | --- | --- | --- | --- | --- | --- | --- |
|  | AP  (n= 60424) | C  (n=302120) | AP | C | AP | C | AP | C | AP | C | AP | C | AP  (n=647) | C  (n=5968) |
| Smoking, n (%) | NR | NR | NR | NR | NR | NR | NR | NR | NR | NR | NR | NR | 330 (51) | 2957 (49.5) |
| Alcohol intake, n (%) | NR | NR | NR | NR | NR | NR | NR | NR | NR | NR | NR | NR | 538 (82.2) | 4728 (76.3) |
| Obesity, n (%) | NR | NR | NR | NR | NR | NR | NR | NR | NR | NR | NR | NR | NR | NR |
| Dyslipidaemia, n (%) | 4478 (7.4) | 16181 (5.4) | NR | NR | NR | NR | NR | NR | NR | NR | NR | NR | NR | NR |
| Hypertension, n (%) | 14752 (24.4) | 50243 (16.6) | NR | NR | NR | NR | NR | NR | NR | NR | NR | NR | 260 (40.2) | 2178 (36.5) |
| Diabetes (type 1 or 2), n (%) | 6820 (12.2) | 23392 (7.8) | NR | NR | NR | NR | NR | NR | NR | NR | NR | NR | 95 (14.7) | 728 (12.2) |
| Chronic kidney disease, n (%) | 925 (1.5) | 4653 (1.5) | NR | NR | NR | NR | NR | NR | NR | NR | NR | NR | NR | NR |
| Coronary artery disease, n (%) | 7291 (12.1) | 24149 (8.0) | NR | NR | NR | NR | NR | NR | NR | NR | NR | NR | 0 (0) | 0 (0) |
| Valvular heart disease, n (%) | NR | NR | NR | NR | NR | NR | NR | NR | NR | NR | NR | NR | 0 (0) | 0 (0) |
| Congenital heart disease, n (%) | NR | NR | NR | NR | NR | NR | NR | NR | NR | NR | NR | NR | 0 (0) | 0 (0) |
| Previous cardiac surgery, n (%) | 360 (0.6) CABG | 1859 (0.6) CABG | NR | NR | NR | NR | NR | NR | NR | NR | NR | NR | 0 (0) | 0 (0) |
| Heart failure, n (%) | 1879 (3.1) | 7617 (2.5) | NR | NR | NR | NR | NR | NR | NR | NR | NR | NR | 0 (0) | 0 (0) |

Abbreviations: AP, asthma patients; C, controls; CABG, coronary artery bypass graft; NR, not reported

1. Patients included were free of cardiovascular disease at baseline

**Supplementary Table 3. Severity and therapy of asthma patients**

|  | **Carter 2019** | **Cepelis 2018** | **Chamberlain 2018** | **Chan 2014** | **Jani 2018** | **Martin-Perez 2016** | **Tattersall 2020** |
| --- | --- | --- | --- | --- | --- | --- | --- |
| **Asthma diagnosis** | ICD-10/OPCD-4 coding systems | Self-reported asthma diagnosed by a doctor | Electronically retrieving diagnostic codes | ICD code 493.xx | Self-reported, touch screen and nurse-led questionnaire | Read classification code | Self-reported physician-diagnosed |
| **Asthma severity** | NR | There was a dose-response association between levels of asthma control and risk of AF with the highest risk for AF in participants with uncontrolled asthma (adjusted hazard ratio, 1.74 [95%CI, 1.26–2.42]; *P* for trend < .001) | NR | NR | NR | NR | Persistent asthmatics had a greater risk of incident  AF (hazard ratio, 1.49 [95% CI, 1.03–2.14], *P*=0.03) |
| **Corticotherapy** | NR | NR | NR | Corticosteroid use has been demonstrated to be associated with an increased risk for AF (OR, 2.13; 95% CI, 1.226–3.701, p= 0.007) | NR | NR | NR |
| **Beta 2 – agonists therapy** | NR | NR | NR | Non-corticosteroid bronchodilator treatment  was associated with an increased risk of developing AF (OR, 2.849; 95% CI, 2.48–3.273, P <0.001) | NR | NR | NR |

Abbreviations: ICD-10 International Classification of Disease 10^th^ edition; OPCD-4 Office of Population Censuses and Surveys Classification of Interventions and Procedures; NR not reported

**Supplementary Table 4. Atrial Fibrillation Outcome**

| Authors, year of publication | Outcome adjustment | Odds Ratio (95% CI) |
| --- | --- | --- |
| Carter, 2019 | Age, gender, ethnic group, cardiovascular disease, cardiac procedures, and common causes of death | 1.016 (0.969-1.066) |
| Cepelis, 2018 | Alcohol use, physical activity, education level, waist-to-hip ratio, diabetes mellitus | 1.38 (1.18-1.61) |
| Chamberlain,2017 | Obesity, smoking, hypertension, CHF, CAD, hyperlipidemia, stroke, arthritis, cancer, CKD, COPD, dementia, depression, diabetes, osteoporosis, schizophrenia, substance abuse, anxiety | 0.84 (0.63 – 1.11) |
| Chan, 2914 | Age, gender, hypertension, diabetes, CHF, CAD, CKD, use of inhaled corticosteroids, oral corticosteroids, and bronchodilators | 1.2, 9 (1.109–1.298) |
| Jani, 2018 | Age, gender,  socio-economic status, smoking, and anticoagulation status | 1.46 (1.02-2.08); 0.03 |
| Martín-Pérez 2016 | Age, gender, time interval between start date and index date, and primary care practitioner visits in the year before the index date | 1.23 (1.05–1.43) |
| Tattersall 2020 | NR | 1.04 (0.82-1.31) |

Abbreviations: CAD, coronary artery disease; CHF, chronic heart failure; CI, confidence interval; CKD, chronic kidney disease; COPD, chronic obstructive pulmonary disease; NR, not reported


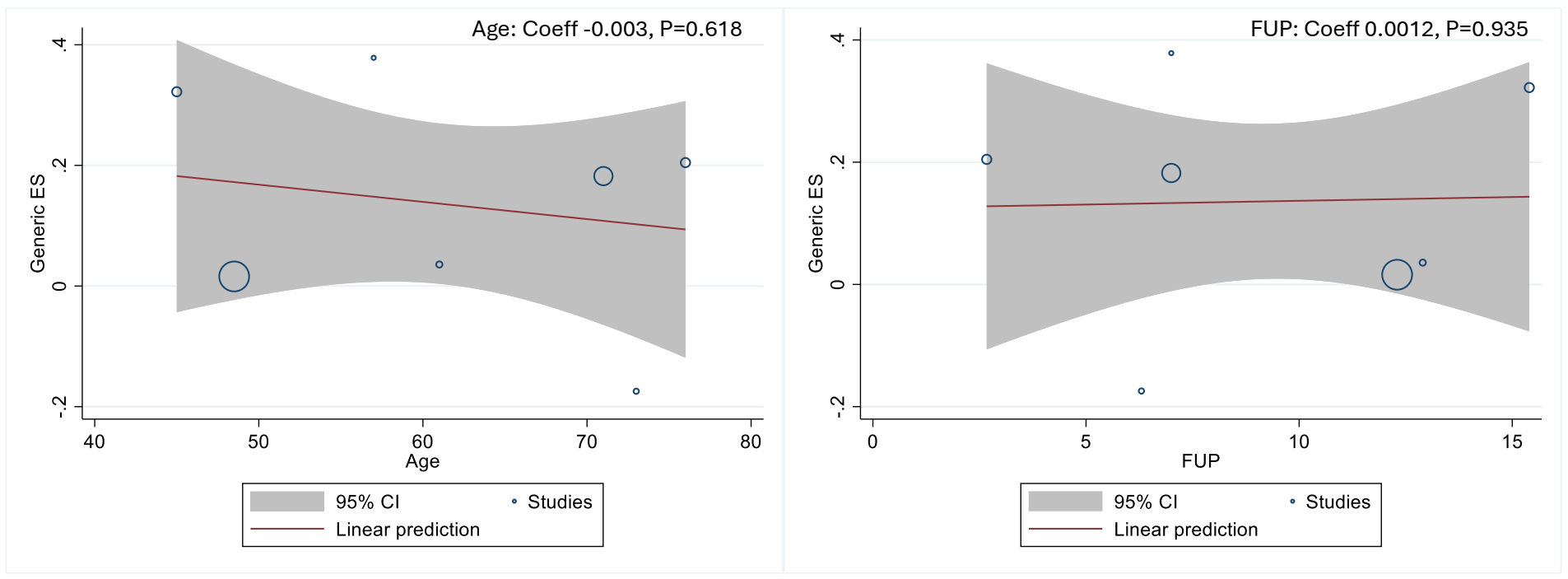


**Supplementary Figure 1: results of the metaregression against age (left) and follow-up time (right).**

**Supplementary Figure 2: Funnel plot for the AF risk in patients with asthma.**

**Supplementary Table 5. Summary of findings according to GRADE criteria (Grading of Recommendation, Assessment, Development and Evaluation).**

| Outcomes | Relative effect (95% CI) | № of studies | Certainty of the evidence (GRADE) | Comments |
| --- | --- | --- | --- | --- |
| Atrial Fibrillation | **OR 1.15** (1.03 to 1.42) | 7 observational studies | ⨁◯◯◯ Very low^a,b^ | Asthma may increase risk atrial fibrillation, but the evidence is very uncertain. |

*Explanations*

a. Overall risk of bias was classified as "serious" for all studies

b. High heterogeneity was detected (I2 = 81%)
